# Expanding a dynamic flux balance model of yeast fermentation to genome-scale

**DOI:** 10.1186/1752-0509-5-75

**Published:** 2011-05-19

**Authors:** Felipe A Vargas, Francisco Pizarro, J Ricardo Pérez-Correa, Eduardo Agosin

**Affiliations:** 1 Department of Chemical and Bioprocess Engineering, School of Engineering, Pontificia Universidad Católica de Chile, Casilla 306 - Correo 22, Santiago CHILE

## Abstract

**Background:**

Yeast is considered to be a workhorse of the biotechnology industry for the production of many value-added chemicals, alcoholic beverages and biofuels. Optimization of the fermentation is a challenging task that greatly benefits from dynamic models able to accurately describe and predict the fermentation profile and resulting products under different genetic and environmental conditions. In this article, we developed and validated a genome-scale dynamic flux balance model, using experimentally determined kinetic constraints.

**Results:**

Appropriate equations for maintenance, biomass composition, anaerobic metabolism and nutrient uptake are key to improve model performance, especially for predicting glycerol and ethanol synthesis. Prediction profiles of synthesis and consumption of the main metabolites involved in alcoholic fermentation closely agreed with experimental data obtained from numerous lab and industrial fermentations under different environmental conditions. Finally, fermentation simulations of genetically engineered yeasts closely reproduced previously reported experimental results regarding final concentrations of the main fermentation products such as ethanol and glycerol.

**Conclusion:**

A useful tool to describe, understand and predict metabolite production in batch yeast cultures was developed. The resulting model, if used wisely, could help to search for new metabolic engineering strategies to manage ethanol content in batch fermentations.

## Background

Management of ethanol yields is emerging as one of the most relevant challenges for biotechnology, including both ethanol maximization (*i.e*. bioethanol and distilled beverages industries) and reduction/minimization (*i.e*. wine, bakery and commodities industries). Significant advances have been made in modeling ethanol fermentations [1-5], although steady-state, gene-modification strategies have resulted in varying degrees of success (for review see [6-8]) mainly due to growth impairment. In turn, dynamic models are able to describe and predict batch fermentations' time-courses better, since different metabolic stages are considered.

A widely used modeling approach to predict cell behavior beyond calibration data is Flux Balance Analysis (FBA), which represents cell biochemical networks as a set of underdetermined constrained mass-balances. In this framework, linear programming is applied to generate a flux distribution that optimizes a given objective function, subject to flux balance equations and constraints. Objective functions commonly used are maximization of ATP production [9,10], maximization of biomass formation [11-13], minimization of metabolic adjustment (MoMa) [14] or minimization of ATP consumption [15,16]. So far, growth maximization has been the most extensively used approach to describe the physiology during growth.

Applying FBA in large-scale metabolic reconstructions, termed Genome-Scale metabolic models (GSMM), has allowed establishing a direct relationship between genetic data and metabolic activity fluxes. These models have been shown to be very useful in predicting the physiological behavior of a microorganism under different genetic and environmental disturbances, *i.e*. growth rate and product secretion patterns [11,13,17-19]. GSMM can help to speed up the design of cells with improved and desired properties, providing metabolic engineering targets that are experimentally testable [20-22]. For example, bacterial genome-scale models have been used to design strains that overproduce lycopene [23], lactate [24], succinate [25], 1,3-propanediol [26], hydrogen [20], L-valine [27] and L-threonine [28]. In turn, genome-scale yeast models have been mainly applied to design ethanol overproducing strains [22,29-31]; nevertheless, design of ethanol underproducing strains have been overlooked, a key issue in wine industry today [5,32-34].

Many computational tools for identifying strain modifications leading to targeted overproductions have been described in the literature. One of the earliest efforts was the OptKnock [26] procedure that proposed gene knockouts leading to targeted overproductions. Later, OptReg [35] expanded OptKnock to consider not only knockouts but also overexpressions and down regulations. In addition, OptStrain [20] allowed for knock-ins of non-native genes to enable production of desired biochemicals. Evolutionary search procedures for solving the resulting combinatorial optimization problems were explored in OptGene [36]. Recently, OptForce [21] was used to identify flux manipulation leading to targeted overproductions. However, strain optimization requires taking into account the whole bioprocess, *i.e*. growth as well as non-growth periods, such as lag, log and steady-state phases. Furthermore, strain optimization needs microbial metabolic modeling expanded upon constraint-based FBA that incorporates experimental validation [37], which has not always been considered in GSMM development. Including these issues will improve the capability of the models to predict the impact of several environmental and genomic alterations on the course of batch fermentations.

Several yeast genome-scale metabolic models have been described so far: iFF708, iND750, iLL672, iIN800, iMM904 and Yeast 4.0 [18,19,38-41]. Despite their advantages, all of them can only simulate time-invariant extracellular conditions consistent with continuous culture, but are not able to reproduce features of the microbial growth process. Dynamic FBA models are increasingly applied to simulate bacteria [42-45], yeast [15,46,47], plant [48] and animal [49,50] growth under several conditions [51,52]. However, the application of dynamic genome-scale FBA models for yeast [29,30] and bacteria [52-54] has been barely explored, and only one bacterial dynamic GSMM model has been experimentally validated.

Over the past few years, our group has been developing a dynamic FBA model (DFBA) to represent anaerobic *S. cerevisiae *batch fermentations [15,47]. This model, comprising a reduced stoichiometric network (39 metabolic reactions), accurately described fermentation profiles under different environmental conditions. However, the lack of detailed metabolic description makes it unsuitable for metabolic engineering studies. To overcome this limitation, we expanded our previous DFBA model of *S. cerevisiae *metabolism [15,47] to genome-scale. This model included kinetic expressions to dynamically constrain the uptake of nutrients, biomass and maintenance. In addition, key improvements related to anaerobic metabolism were identified. Furthermore, we carried out a comprehensive validation using experimental profiles of batch fermentations and final concentration of metabolic products under genetic/environmental disturbances. To the best of our knowledge, we report here the first experimentally validated GS-DFBA for *S. cerevisiae*.

## Methods

### Model formulation

**Figure 1:**
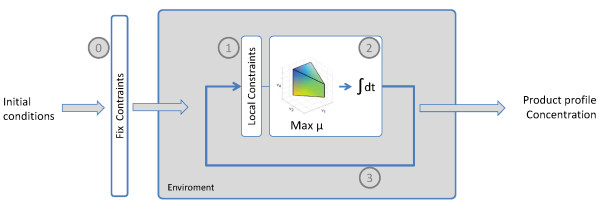
**Resolution algorithm of idFV715 model**. The model is based on an iterative optimization of an under determined matrix, using LINDO optimization software (LINDO system): 0) Fixed constraints to be used throughout fermentation are defined, such as genetic background or nutritional requirements; 1) Dynamic constraints are defined as bounds fluxes set to by intracellular extracellular conditions; 2) LP solves the metabolic flux distribution, as well as the consumption and production rates, at 30 min intervals; 3) The resulting rates are used as inputs for the differential equations solved using a variable step integration routine.

The current version of our genome-scale dynamic-flux-balance-analysis model (GS-DFBA) consists of four interacting blocks, which are solved sequentially (Figure 1). First, initial conditions and fixed constraints are specified (block 0). Next, dynamic constraints which depend on metabolite concentrations in the medium are established (block 1). A linear programming (LP) problem is then solved (block 2) to compute the consumption and production rates of extracellular metabolites. Finally, these production rates are transferred to an ODE solver (block 3) that integrates the respective bioreactor mass balances during intervals of 30 minutes (keeping extracellular rates constant), providing accurate results without losing fermentation details [15,47]. A sensitivity analysis showed small variations when this time interval is changed (Additional file 1). The procedure iterates between blocks 1, 2 and 3 until the sugar is consumed or an unfeasible condition is found.

Typical initial conditions that must be defined *a priori *are temperature, and the concentration of nitrogen-compounds, sugar-compounds, extracellular metabolites and biomass. The LP is bounded by fixed and dynamic constraints. Fixed constraints include carbon/nitrogen limiting medium, oxygen presence or absence, biomass equation, and genetic background. Dynamic constraints include sugar/nitrogen uptake kinetics, maintenance and carbohydrate accumulation. Following this procedure, it takes 43 s of CPU time to simulate a normal alcoholic fermentation (200 g/L of sugars and 300 mg/L of assimilable nitrogen) in a Core2Duo 2.66 GHz processor. The features of each block are detailed below (Figure 1).

#### Linear programming

A standard FBA model [13,55,56] comprises an underdetermined metabolic network of s molecular species and *v *reactions, which is represented by a *v *× s stoichiometric matrix (T). To solve the intracellular fluxes as well as the consumption and production rates of metabolites in the cell, an LP is defined (eq. 1), where an objective function for cell metabolism *v*_j _should be specified. Following common practice, maximization of growth rate was used to mimic growth in exponential phase [11-13]; however, when the limiting nutrient is exhausted, minimization of ATP consumption by maintenance was applied [15,16].(1)

where *v *is the vector of metabolic fluxes, and  and  are dynamic lower and upper bounds of the flux *i*, respectively. All LP calculations were carried out within MATLAB (The MathWorks Inc.) using Lindo optimization package (Lindo Systems Inc.).

To reduce the impact of multiple optimal solutions that normally appear in FBA [57], for metabolic engineering approaches we carried out a bi-level optimization procedure [29], solving first the LP for maximum biomass, and then fixing the biomass at this maximum value and solving the LP again to optimize a second objective (*i.e*. maximizing/minimizing ethanol).

Up until now, several yeast genome-scale model databases have been published: iFF708 [18], iND750 [19], iLL672 [40], iIN800 [39], iMM904 [38] and Yeast 4.0 [41]; each one adding more details to the metabolic network. However, additional information regarding compartmentalization, cycles and expansion of previously lumped reactions has not necessarily led to improved accuracy and better predictions.

The first published genome-scale model, iFF708, was the simplest and has good predictive performance. Therefore, The GS-DFBA model described here was developed using an updated version of the iFF708 stoichiometric matrix [18] coupled with our previously developed algorithms of DFBA (Figure 1) [15,47]. The resulting model, idFV715, where "d" stands for dynamic and "i" for *in silico*, consists of 715 structural ORF's that catalyze 705 distinct biochemical reactions and 145 putative reactions that have not been associated with any gene yet, comprising 590 metabolites and 1181 metabolic reactions in total. Compared with iFF708, the idFV715 model includes a more detailed description of flavor production related to acetoin and butanediol syntheses.

#### Dynamic mass balance

During microbial growth, the environmental conditions are changing constantly due to the consumption of nutrients and secretion of metabolites. These changes are modeled by the following set of ordinary differential equations:(2)

Where *M_EX _*is the vector of concentrations of extracellular metabolites in the medium, *V_EX _*is the vector of the respective specific consumption and production rates, and *X_V _*is the concentration of viable biomass in the medium.

#### Fixed constraints

These include all constraints that remain constant during the whole fermentation, such as genetic background (deletions, insertions and overexpressions), carbon/nitrogen limiting medium that modify the biomass equation, and lack of oxygen that modify oxygen-related reactions.

As yeast responds differently to aerobic or anaerobic conditions [58-60] we described the *S. cerevisiae *physiology under these conditions as closely as possible. The expression of approximately one hundred genes is regulated by oxygen availability [61,62], affecting mainly the cell redox state [63]. This, in turn, affects ATP, biomass and product syntheses. For instance, approximately eight times less ATP and ten times less biomass are produced under anaerobic conditions [64,65].

In our approach, we bounded to zero the oxygen uptake-related flux. In addition, we included a known transcriptional regulation, *i.e*. during anaerobic fermentation the TCA cycle has been shown to be broken at the level of succinyl CoA synthetase, acting as two separate branches - the "oxidative branch" and the "reductive branch." This behavior was first established as thermodynamically feasible in *S. cerevisiae *[66], and then supported by Metabolic Flux Analysis (MFA) [67]. Later, Camarasa et al. [68] experimentally confirmed it, using NMR isotopic filiation. Hence, the genes involved in succinate dehydrogenase complex and succinyl-CoA ligase were bound to zero, thus forcing the TCA cycle to act as two branches. Also, quinone mediated reactions involving FADH_2 _and NADH reoxidation were turned off. Finally, ergosterol, lanosterol and zymosterol upper bound constraints, UB, were set to infinity (assumed to be non-limiting) in anaerobic conditions [69-71].

Furthermore, to ensure the presence of inorganic compounds in every condition studied, phosphate and sulphate concentration in the medium were also set to infinity (UB assumed to be non-limiting).

#### Dynamic constraints

Sugar transport is the main rate-limiting step in N-limited fermentations [72]. Known regulatory effects have been previously used by our group to include sugar uptake expressions into a DFBA model [1]. The efficiency of the uptake of sugars is altered by the gene expression, where a set of specific hexose transporters (HXT) are expressed in response to environmental signals [73,74]. Moreover, these transporters show competitive inhibition between glucose and fructose, and their activity is modulated by temperature through changes in the activation energy of the process as well as by non-competitive inhibition by ethanol [75]. Kinetic expressions that considered all these effects were incorporated as UB (upper bound) into the idFV715 model (Additional file 2: eqs. 1, 2).

An empirical function was developed to describe total YAN (Yeast Assimilable Nitrogen) consumption. We incorporated normalized time-varying fluxes for each nitrogen-compound (amino acids/ammonia) into a previously reported equation for total nitrogen consumption [15,47], which was derived from an experimental lookup-table using TableCurve 2D software (Systat Software Inc.). The uptake flux of each N-compound changes according to total nitrogen concentration in the medium, representing a competition for the limited number of nitrogen transporters. These fluxes were included as UB constraints in the LP (Additional files 2: eq. 3).

Yeast faces changing conditions during fermentation, triggering metabolic stress responses and increasing ATP consumption by maintenance (m_ATP_) [34,76,77], as determined in media with ethanol concentrations above 4% v/v [78]. High temperatures also result in increased cellular maintenance, to restore the membrane potential lost by ion diffusion and from altered protein synthesis [79,80]. In addition, sluggish fermentations arising from nitrogen deficiency show higher maintenance costs than normal fermentations [81]. Therefore, to adequately model yeast response to stress, a maintenance term was included that estimate the specific energy requirements to maintain cellular function during fermentation. A lower bound to m_ATP _consumption is set by an empirical function that assigns maintenance costs according to fermentation conditions [47] (Additional file 2: eq. 4). This function, fitted from an experimental lookup-table using TableCurve 2D software, represents the influence of ethanol, high temperature and other energy expenditures associated with sluggish cultures [15].

Accurate simulation of the growth rate in FBA models requires a careful formulation of the biomass equation [38-40,82]. This should reflect observations that biomass composition changes with environmental conditions and as fermentation progresses [39,81,82]. Hence, we used a biomass equation that considered new data and experimentally observed variations in the biomass composition of *S. cerevisiae *under different growth conditions. This medium-specific biomass equation was reported in iIN800 model [39] (Additional file 2). Our approach incorporates an empirical carbohydrates accumulation expression (Additional file 2: eq. 5)[15] to the medium-specific biomass equation [39] to give time-dependent behavior. The equation describing the accumulation of carbohydrates was determined by fitting measured rates to sugar consumption [15]. This function defines an UB in the LP. This final equation is referred to in this article as time-medium-specific biomass equation.

### Model validation

#### Experimental fermentations

idFV715 was validated with data obtained in the laboratory as well as from industrial fermentations. Laboratory data comprised 12 fermentations carried out by our group [15,79] and 8 fermentations provided by the Sciences pour l'Oenologie Research Unit, INRA, Montpellier, France (Drs. Sylvie Dequin and Carole Camarasa, unpublished). Both datasets correspond to alcoholic fermentations performed at several temperatures (20-34°C) under different initial carbon (150-308 g sugar/L) and nitrogen (50-538 mg N/L) concentrations [15,79]. All were carried out in 1 L bioreactors using *S. cerevisiae *EC1118 or V5 wine strains.

Industrial data come from 10 anisothermic wine fermentations with different initial sugar concentrations (181-250 g/L) [79]. These were performed during the 2003 vintage in Chile; in fermentation tanks of 40 to 60 m^3 ^of Cabernet Sauvignon inoculated with *S. cerevisiae *EC1118 strain.

#### In silico genetic modifications

The impact of the gene modifications on simulations of batch fermentations, as measured in final product concentrations, was compared with literature data. To simulate the effect of a single gene deletion, the fluxes through the corresponding reactions were constrained to zero during the whole fermentation [13,42]. *In silico *detrimental gene deletions lead to lower growth rates compared to wild-type simulations, yielding lower biomass and/or longer fermentation times.

We assessed the predictive performance of the idFV715 model by comparing simulation results with 35 different environmental and genetic modifications reported in the literature. These include overexpressions [5,83-85], deletions [5,83,84,86-89], insertions [90] and cofactor engineering [5] in single, double and triple mutant strains (Additional file 3). The "R" score was used to assess model performance:(3)

This expression compares experiments and simulations in terms of the relative changes in the final concentration of given metabolites. Here, D is the modeling (M) or experimental (E) concentration of the metabolite "i" when it is genetically modified (GM) or wild type (WT). Hence, values of R close to zero mean good model predictions.

Fermentation conditions that were not given in the literature were estimated to reproduce the respective experimental results as closely as possible. For example, environmental conditions such as temperature, oxygen, sugars, amino acids, ammonia, ethanol and glycerol, were normally given in the literature, but vitamins and inorganic compounds of the medium were not. In addition, deletions and insertions were well described in the literature references; however, the levels of overexpression were not given. Therefore, due to the nature and diversity of information sources, estimating fermentation conditions was often difficult and an additional source of error.

## Results and discussion

### Expanding DFBA to genome-scale

In this study, we expanded a small and reliable 39-equation DFBA model of *S. cerevisiae *metabolism [15,47] to genome-scale. Our aim was to obtain increased insight into yeast metabolism (given by the genome-scale), while maintaining simulation performance of the previous DFBA model. Therefore, we built a GS-DFBA (idFV715) that accurately predicts the synthesis and consumption profiles of yeast metabolites during the alcoholic fermentation.

To assess the new model, first we analyzed the impact on simulation performance of key model improvements. Then, we validated model results with experimental data.

#### Assessment of model improvements

Proper choice of the biomass equation is normally considered a critical component in FBA-based modeling [38-40,82], although adequate handling of ATP maintenance and anaerobic conditions are also key issues that have been overlooked so far. In this study, we explore the impact on the idFV715 model's predictions of maintenance, biomass and anaerobic constraints.

##### Maintenance

Fermentations were simulated anaerobically in N-limiting medium; the variables were low and high temperatures, as well as sluggish and normal conditions. In each case, four maintenance models were assessed: i) mATP constrained according to eq. (4) (Additional file 2), ii) mATP constrained to zero, iii) unconstrained mATP, and iv) mATP constrained to a typical value used in previous models (see Table 1).

**Table 1:** Assessment of maintenance term used in idFV715

Initial conditions							
Temperature [°C]	Nitrogen [mg/L]	Sugars [g/L]		Time (H)	Biomass (g/L)	Glycerol (g/L)	Ethanol (g/L)
			Experimental wt	455	4.65	ND	ND
			Model with maintenance	422	4.97	-	-
12	300	268	Model maintenance constrained to zero	415	5.00	-	-
			Model maintenance unbound	451	5.00	-	-
			idFV715 Model using iFF708 maintenance term	489	4.79	-	-
			Experimental wt	700	1.49 ± 0.47	10.98 ± 0.28	75.41 ± 6.50
			Model with maintenance	710	0.93	9.95	96.85
28	50	238	Model maintenance constrained to zero	683	0.97	1.18	99.84
			Model maintenance unbound	748	0.97	1.24	117.24
			idFV715 Model using iFF708 maintenance term	784	0.92	1.71	118.90
			Experimental wt	122	5.38 ± 0.43	7.93 ± 0.28	107 ± 3.52
			Model with maintenance	131	5.14	10.02	106.54
28	300	233	Model maintenance constrained to zero	134	5.19	4.37	113.23
			Model maintenance unbound	132	5.19	15.57	106.73
			idFV715 Model using iFF708 maintenance term	133	5.12	3.47	112.60

Simulations assessing the cost of maintenance in three different environmental conditions. ND means no determined product concentration. Each experiment represents average values from three independent replicates, except for low-temperature conditions.

The most significant difference among model simulations was glycerol prediction. When maintenance flux is constrained according to our maintenance equation (Additional file 2: eq. 4), glycerol predictions are much closer to measured values than the other simulations. In addition, simulations with unbounded or zero maintenance tend to overpredict biomass concentrations, partially explaining the impaired results on glycerol synthesis obtained in these simulations. This is probably due to new environmental conditions where maximization of the growth rate resulted in minimizing ATP costs from glycerol synthesis. Furthermore, when simulations were constrained to a commonly used value from previous genome-scale models (*i.e*. 1 mmol/g DW h^-1^) [18,63], the ATP maintenance expenditure is fixed with a higher value than the output of the maintenance equation proposed in this paper. This higher maintenance cost results in lower biomass and glycerol synthesis and hence in longer fermentation times.

Ethanol prediction also showed significant differences among models. A decrease in glycerol synthesis results in increased carbon fluxes through glycolysis. Hence, unbounded, zero maintenance and constrained-to-previous-value simulations result in higher ethanol contents than those obtained with our maintenance equation (Additional file 2: eq. 4).

Moreover, the latter predicts ethanol concentrations closer to experimental data. The model with our maintenance equation tends to overestimate ethanol synthesis in the nitrogen-lean condition though. To grow under stress, yeasts might divert the carbon flux to other metabolic pathways not accounted for in our model, such as synthesis of lipids and cell-wall precursors. Therefore, a careful examination of these components could be useful to improve model results.

##### Biomass composition

Since biomass composition varies throughout fermentation and depends on media composition, a time-medium-specific biomass expression was included (see methods and Additional file 2). To assess the impact on model predictions of this biomass expression, two simulations were carried out using i) current biomass expression; and ii) iFF708 biomass expression (Table 2).

**Table 2:** Assessment of biomass expression used in idFV715

Initial conditions					
Temperature [°C]	Nitrogen [mg/L]	Sugars [g/K]		Time (H)	Biomass (g/L)
			Experimental wt	455	4.65
12	300	268	idFV715 Model	422	4.97
			idFV715 Model using iFF708 biomass eq.	557	3.84
			Experimental wt	700	1.49 ± 0.43
28	50	238	idFV715 Model	0.93	
			idFV715 Model using iFF708 biomass eq.	0.78	
			Experimental wt	122	5.38 ± 0.43
28	300	233	idFV715 Model	131	5.14
			idFV715 Model using iFF708 biomass eq.	154	3.99

Assessing the impact of different biomass expressions in three different environmental conditions. The "idFV715 model" uses the updated time-medium-specific biomass expression. The "idFV715 model using iFF708" includes the biomass expression of iFF708. Each experiment represents average values from three independent replicates, except for low-temperature conditions.

The updated time-dependent biomass expression developed in this work significantly improved biomass predictions compared to simulations using the iFF708 biomass expression. In the first case, using experimental data, an average error ranging from 0.2-0.6 g/L was found. In turn, iFF708 biomass calculations with the GS-DFBA showed deviations between 0.7-1.4 g/L. In both cases, better performances were accomplished in fermentations with standard nitrogen levels (300 mg/L YAN), compared to lean-nitrogen cultures. Underestimation of cell growth under low-nitrogen conditions strongly suggests that cells maximize the use of nitrogen compounds under nutrient limitation, a fact that is not accounted in idFV715.

##### Anaerobic conditions

To assess the impact of anaerobic constraints on model predictions, we performed simulations by varying the activation or inhibition of the following specific constraints: i) oxygen uptake constrained to zero, ii) unconstrained sterol and unsatured fatty acids uptake, iii) TCA cycle split up into two branches, and iv) quinone-mediated reactions constrained to zero (Table 3).

**Table 3:** Assessment of anaerobic constraints used in idFV715

	Oxygen uptake	Sterols uptake**	Quinone reactions	Complete TCA cycle*	Time (H)	Biomass (g/L)	Glycerol (g/L)	Ethanol (g/L)
Experimental wt	-	-	-	-	122.0	5.38 ± 0.43	7.93 ± 0.282	107 ± 3.52
**Model 1 (Used as wt)**	**off**	**unlimited**	**off**	**off**	**130.50**	**5.14**	**10.02**	**106.54**
Model 2	off	unlimited	on	off	137.50	5.10	1.50	115.07
Model 3	off	unlimited	off	on	129.50	5.50	1.66	115.21
Model 4	off	unlimited	on	on	137.50	5.09	2.14	114.35
Model 5	on	unlimited	off	off	111.50	5.49	29.44	90.44
Model 6	off	limited	off	off	L	L	L	L

Assessing the impact of different factors in anaerobic metabolism. Fermentations were carried out in triplicate in the nitrogen-limited medium MS300 at 28°C. Different models represent simulations by varying the activation or inhibition of the specific constraints. L = lethal; * Complete TCA cycle means no branches. **Sterols uptake means ergosterol and zymosterol uptake.

L = lethal

*Complete TCA cycle means no branches

** Sterols uptake mean ergosterol and zymosterol uptake

Simulations in the absence of oxygen were only feasible when the availability of unsaturated fatty acids and sterols is not constrained (Table 3: model 6). Since *S. cerevisiae *cannot synthesize these compounds (ergosterol and zymosterol) under anaerobic conditions [69-71], this constraint is usually considered in anaerobic models [29,30].

However, restrictions to TCA and quinone-reactions have received little attention and few studies have incorporated transcriptional regulatory information to respond to these environmental conditions [91]. We found that TCA constraints are essential to describe the two separate branches operating under anaerobic conditions - the "oxidative branch" and the "reductive branch" [66-68], which are critical to get accurate predictions. In this condition, glycerol and ethanol synthesis compared very well with experimental data (Table 3: models 1, 3 and 4). When the TCA cycle is unconstrained, glycerol synthesis decreases and ethanol increases. This is probably due to the TCA cycle working as a reducing cycle under these unconstrained conditions, reducing the excess NADH. Consequently, glycerol synthesis would be no longer needed, increasing carbon flux through glycolysis and so, ethanol synthesis.

In addition, results showed that quinone-mediated oxidations of FADH_2 _and NADH play a fundamental role in aerobiosis (not shown); however, under anaerobiosis their effect worsens model predictions. When these reactions are turned off, predictions of fermentation time, biomass, glycerol, and ethanol synthesis are closer to measured values (Table 3: models 2, 4). This is because in idFV715, quinone-mediated reactions do not require oxygen as a precursor, since they always act as electron acceptors, replacing the redox function of glycerol synthesis. Consequently, the rate of ethanol synthesis increases due to a higher carbon flux in glycolysis.

In summary, a significant improvement in glycerol, ethanol, biomass and fermentation time prediction were achieved when the model included the full set of anaerobic constraints described above, in addition to the new maintenance and biomass equations.

#### Model validation

We considered for validation 2 types of published experimental data: i) batch fermentation kinetics; and ii) final concentration of metabolic products under genetic/environmental disturbances. These analyses highlight the differences with our previous model while maintaining model performances.

##### Fermentation profiles

Two sets of batch fermentations with *wt *yeasts were used. The first set corresponded to 20 laboratory fermentations under different temperature and initial concentrations of nitrogen and sugars. Ten industrial wine fermentations with different initial glucose concentrations and a time varying temperature profile comprised the second set.

**Figure 2:**
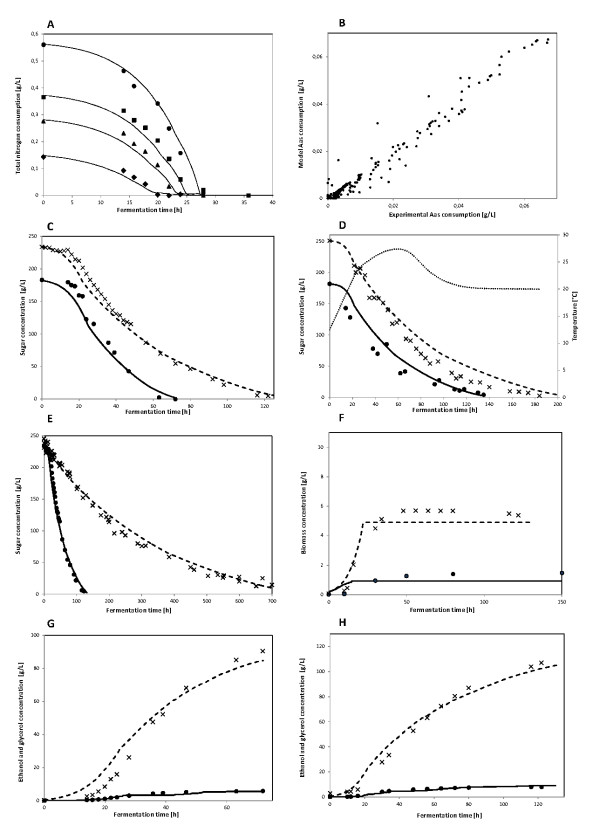
**idFV715 model performance**. Model predictions of consumption and production rates of the main metabolites and nutrients involved in an alcoholic fermentation. In this figure, symbols represent the experimental data and lines represent model prediction. Measured values were in triplicate with a CV <5%: A: Experiments and simulations of isothermal, laboratory-scale fermentations, in 4 conditions of total-assimilable-nitrogen (100, 200, 300, 400 mg/L of YAN); B: Experiments and simulations of isothermal, laboratory-scale, high-nitrogen fermentations (300 mg/L of YAN, R = 99%, 400 points), 28°C; C: Experiments and simulations of isothermal, laboratory-scale, high-nitrogen fermentations. Residual sugar concentration at two representative initial conditions of sugar content in the medium (240 and 182 g/L, 300 mg/L YAN, 28°C); D: Experiments and simulations of anisothermal, industrial-scale, high-nitrogen (240 mg/L YAN) fermentations. The fastest and slowest fermentations are shown. Simulations werefile run assuming a typical temperature profile (dotted-line); E: Isothermal, laboratory-scale, residual sugar concentration for the fastest and the slowest fermentation analyzed, corresponding to 50 mg/L YAN and 300 mg/L, respectively; F: Isothermal, laboratory-scale, predicted concentration of biomass under 50 mg/L (closed circles) YAN and 300 mg/L (×) YAN 28°C; G: Isothermal, laboratory-scale, predicted concentration of ethanol (×) and glycerol (closed circles) under 240 g/L sugar, 300 mg/L YAN, 28°C; H: Isothermal, laboratory-scale, predicted concentration of ethanol (×) and glycerol (closed circles) under 182 g/L sugar, 300 mg/L YAN, 28°C.

Table 4 summarizes performance results for idFV715 validation. The numbers in the respective boxes correspond to the correlation, R, between simulation and experimental data for the whole fermentation process. Figure 2 shows consumption and production profiles for main metabolites and nutrients included in Table 4. Sugar prediction at industrial and laboratory scale shows good agreement with experimental data at several initial sugar conditions (Figure 2C, D). Furthermore, sugar prediction shows good correlation at high-nitrogen and low-nitrogen conditions, reflecting standard and sluggish fermentations (Figure 2E). Therefore, for laboratory and industrial fermentations, idFV715 shows a high degree of confidence in sugar uptake profile predictions (glucose and fructose). This step ensures the correct income of energy and carbon to the modeled cell.

**Table 4:** Fermentation profiles prediction of idFV715

Initial Conditions			Lab fermentations				Industrial fermentations
Nitrogen	Sugar	Sugar uptake	Nitrogen uptake	Ethanol	Glycerol	Biomass	Sugar uptake
Low 50-200 mg/L	Low 100-200 g/L	99.5	98.0	99.4	97.7		
	High 201-350 g/L	99.7		99.5	95.4	84.1	
High 201-540 mg/L	Low 100-200 g/L	98.9	98.0	98.6	93.0		97.9
	High 201-350 g/L	99.3	99.0	99.7	98.4	95.1	98.8

Fermentation profiles prediction of idFV715 under different medium conditions. Twenty laboratory and ten industrial fermentations, with approx. 25 samples per fermentation, were analyzed. Performance is expressed as percentage of correlation between experimental and model data.

Nitrogen is usually the limiting nutrient in alcoholic fermentations and it is linked to the redox-balance that eventually determines the synthesis of glycerol, ethanol and acetate, among other metabolites. Despite its relevance, most current models simply define ammonia uptake as the sole nitrogen source or leave amino acid uptake unbounded; only one bacterial GS-DFBA model applied kinetic parameters to define amino acid uptake, although it has not been experimentally validated [53]. Here, we showed that idFV715 with these new nitrogen features accurately predicts nitrogen uptake experimental profiles, *i.e*. ammonia and amino acids kinetics, at several initial nitrogen conditions (see Figure 2A, B and Table 4). This feature is useful to define the correct synthesis of redox-linked metabolites.

Simulated profiles of ethanol and glycerol in laboratory-scale fermentations compare well with experimental data (Figure 2G, H). Moreover, prediction of the biomass profile is in relatively close agreement with experimental data; however they are less accurate at low nitrogen conditions (Figure 2F). This could be partly explained by an experimentally observed delay in biomass synthesis in relation to nitrogen uptake that was not captured by idFV715, and also by a lower biomass prediction at these conditions (See section Assessment of model improvements).

Remarkably, when same data sets were used to compare the idFV715 model to our previous reduced DFBA model [15], on average, performances were quite similar (differences in correlation < 5%) (Additional file 3). Both models show high correlation and a high predictive ability (less than 5% of the real value, with 95% of confidence). Hence, good performance was quite maintained after expansion to the genome-scale.

In summary, despite some differences between model and experimental data, idFV715 predicts relatively well the consumption and synthesis profiles of the main metabolites/nutrients involved in an alcoholic fermentation.

##### Final product concentration

In order to analyze the additional features of the GS-DFBA model, final measured concentrations of metabolic products were compared to idFV715 predictions for 35 fermentations using yeast mutants under a variety of environmental conditions. This set of experiments comprises deletions, overexpressions and insertions in up to three genes (Additional file 3, section 2). These features represent one-step forward compared to our previous model capabilities [15], which had not enough reactions to simulate genetic disturbances.

**Figure 3:**
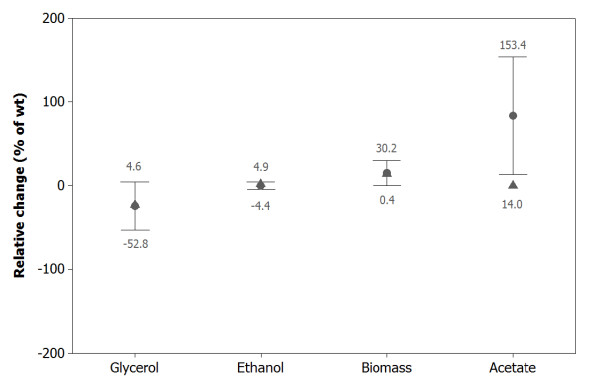
**Prediction error distribution for idFV715**. Prediction error distribution for 35 different yeast fermentations. The errors compare the relative change between model performance and experimental results, under different environmental conditions (considering 95% confidence). Mean (●)and median value of each set (▲).

Analyses of final product concentrations showed that ethanol, glycerol and biomass content *- *the major carbon compounds in alcoholic fermentation - were predicted in close agreement with experimental data (Figure 3). Indeed, ethanol predictions were accurate, with maximum deviations smaller than 5%. Glycerol and biomass synthesis showed an acceptable level of confidence with errors smaller than 50%. In turn, the model often overestimated acetate production. A sensitivity analysis showed that acetate synthesis in the model is highly sensitive to temperature, nitrogen and sugar (Additional file 1). Hence, small errors in these variables (taken from the respective paper or database) can cause large variations in the predicted final acetate concentration. If, in addition, we consider that acetate synthesis represents less than 0.5% of the initial sugar, it is not surprising that idFV715 does not perform well for acetate prediction. Moreover, synthesis and regulation of acetate are not well known, and FBA models include only carbon and redox governed metabolic rearrangements; transcriptional and protein regulations are not taken into account. In a future version of the model, inclusion of some regulatory network could improve these results as other studies have explored [46,91,92].

Instead of predicting exact final concentration values, the model is able to reproduce the relative effects and trend of given genetic engineering strategies on final metabolites concentrations. For example, our model reproduced the experimental results of Guadalupe et al. (2010) [90]. These authors expressed the *E. coli *MHPF gene in *S. cerevisiae*, encoding the NAD-dependent acetaldehyde dehydrogenase to restore redox balance and allow the anaerobic growth of a *S. cerevisiae *Δgpd1/gpd2 strain through acetate consumption. Including these gene modifications, our model predicted an increment of ethanol production of 5%, no glycerol production and consumption of acetate instead of production. Although the model predicts the right trend, the experimental values obtained by Guadalupe et al. (2010) regarding ethanol and acetate were different; they obtained twice the amount of ethanol and almost 7 times more acetate was consumed.

## Conclusions

In this paper, we described the first experimentally validated GS-DFBA model for alcoholic fermentations. Model validation included fermentation profiles and final concentration of fermentation products under different environmental and/or genetic disturbances. We implemented a set of constraints that resulted in key improvements in the physiological response of *S. cerevisiae *under anaerobic fermentation. Here, we showed that not only biomass expression is a critical component of FBA-based modeling but also ATP maintenance and anaerobic constraints. Furthermore, it is noteworthy that a detailed description of nitrogen-compounds uptake helps to improve results. Therefore, under the conditions studied here, idFV715 closely agrees with final concentrations as well as fermentation profiles of the main metabolites involved in alcoholic fermentation, especially ethanol, glycerol and biomass. Minor compounds such as acetate are not well predicted though, mainly due to unaccounted genetic regulations and model sensitivity. A careful examination in the definition of lipids and cell-wall components could help to improve results.

Consequently, idFV715 could be useful to predict the evolution and concentration of the main metabolites from *S. cerevisiae *under different environmental conditions as well as different genetic backgrounds. Given the close predictions of idFV715 regarding ethanol synthesis, this model is especially suitable to design yeast strains for optimum ethanol management in batch fermentations. This model and its future improvements can be used to design new metabolic engineering strategies to optimize fermentation, as well as product synthesis.

## Abbreviations

DFBA: Dynamic Flux Balance Analysis; GSMM: Genome-Scale Metabolic Model; GS-DFBA: Genome-Scale, Dynamic Flux Balance Analysis; MFA: Metabolic Flux Analysis; MoMA: Minimization of Metabolic Adjustments; NMR: Nuclear Magnetic Resonance; LP: Linear Programming; ODE: Ordinary Differential Equation; TCA: Tricarboxylic acid; YAN: Yeast Assimilable Nitrogen.

## Authors' contributions

The authors FAV, FP, JRP and EA, conceived and designed the experiments. FAV performed the experiments and analyzed the data. All authors have read and approved the final manuscript.

## Supplementary Material

Additional file 1 **Sensitivity analyses of the idFV715 model**. This file includes sensitivity analyses of the effect of time step integration and acetate production in the idFV715 model. Click here for file

Additional file 2 **Kinetic expression used in the idFV715 model**. This file includes kinetic expression used in the idFV715 model. Sugar, nitrogen and maintenance expressions are detailed. Click here for file

Additional file 3 **Model validation**. This file includes tables where results using previous DFBA model can be directly compared to current model results of Table 4. Also, a description of metabolic engineering conditions was included. Click here for file
