# Supplementary material for: Expanding a dynamic flux balance model of yeast fermentation to genome-scale
Source: BMC Syst Biol. 2011 May 19;5:75. doi: 10.1186/1752-0509-5-75 (PMC3118138; doi:10.1186/1752-0509-5-75)
Supplement: Additional file 1 — Sensitivity analyses of the idFV715 model. This file includes sensitivity analyses of the effect of time step integration and acetate production in the idFV715 model. [file 1752-0509-5-75-S1.PDF]

Additional File 1

Time Step integration:

To assess the sensitivity of the time step to model predictions, we performed different simulations under different time steps: 0.1, 0.45, 0.5, 0.55 and 1 hour. This represents -80%, -10%, 0%, +10% and +100% with respect to the time step used in the main document (Figure 1).

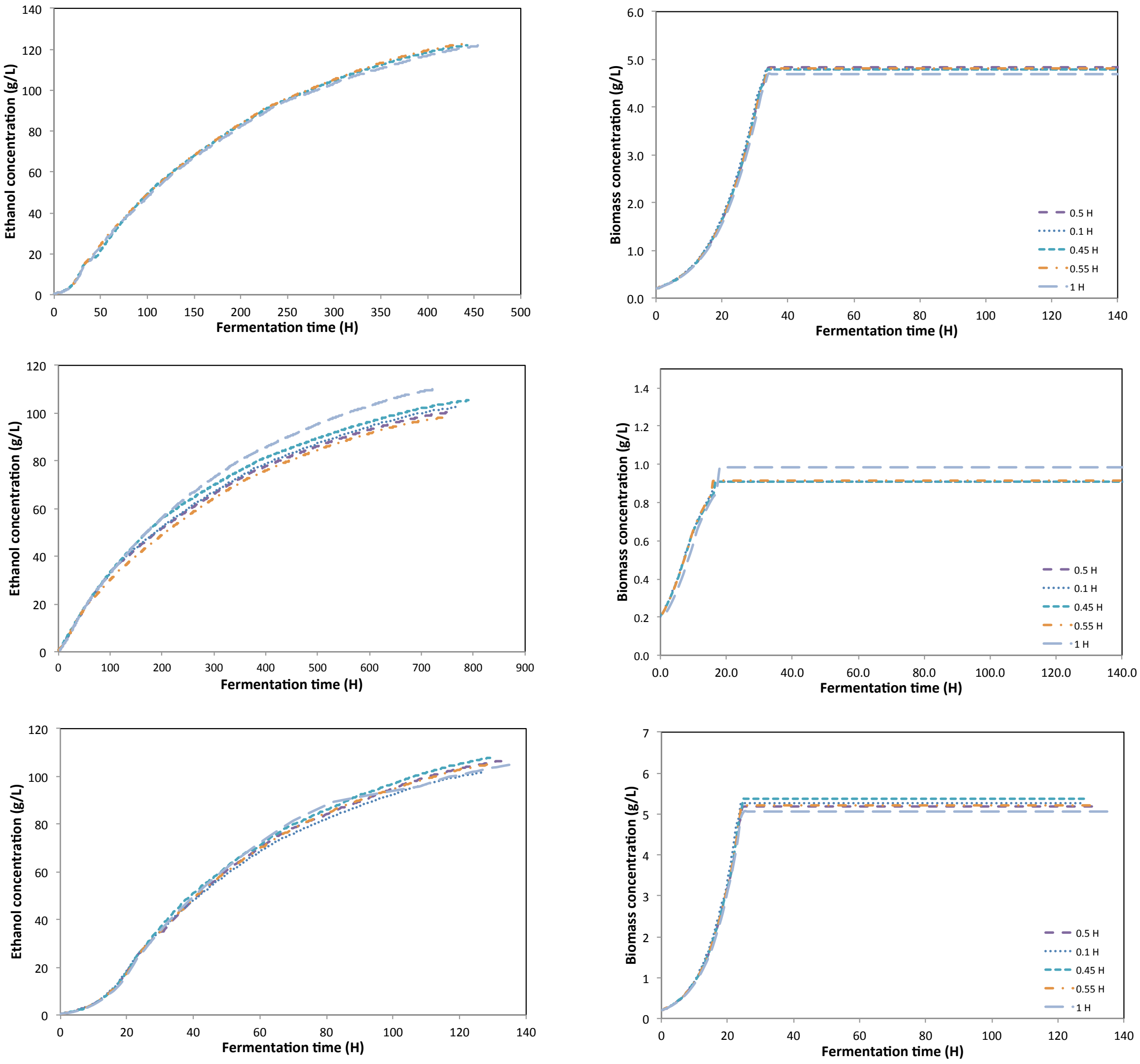

Figure 1: idFV715 simulations under three different conditions using five different time steps of integration: 0.1, 0.45, 0.5, 0.55 and 1 hour. Ethanol (A) and biomass (B) profiles during a 12°C, 300 mg of nitrogen/L and 268 g sugar/L fermentation. Ethanol (C) and biomass (D) profiles during a 28°C, 50 mg of nitrogen/L and 238 g sugar/L fermentation.

Ethanol (E) and biomass (F) profiles during a 28°C, 300 mg of nitrogen/L and 233 g sugar/L fermentation.

To statistically assess the sensitivity of the time step to model predictions, we used the experimental data to calculate the standard error of the estimate (S_{est}), according to the following formula:

$$S_{est} = \sqrt{\frac{\sum_j \sum_i (x_{ij} - \hat{y}_{ij})^2}{N-2}} \quad (1)$$

where x_{ij} is the experimental data observed in the jth experiment at the ith time point and $\hat{y}_{ij}$ is the corresponding model prediction for that time point in that experiment, and N is the total number of experimental points. Small time step variations (±10%) showed standard error inside the 10% variation. The most sensitive time steps are 0.1 and 1 hour because they get away from 0.5 H time step, which corresponds to the value where every parameter in the model was calibrated (Table 1).

Initial conditions			Time step (H)					
			-80%	-10%	0%	10%	100%	900%
Temperature [°C]	Nitrogen [mg/L]	Sugars [g/L]	0.1	0.45	0.5	0.55	1	5
12	300	268	55.00	18.65	24.01	25.11	21.54	40.45
28	50	238	47.05	23.51	20.75	29.39	25.75	75.10
28	300	233	6.36	5.44	6.54	7.19	11.93	13.00

Table 1: Sensitivity assay comparing experimental data with simulation in three different environmental conditions under different time steps 0.1, 0.45, 0.5, 0.55 and 1 hour. Here final values of ethanol, glycerol, biomass and fermentation time were taken into account.

Acetate Production:

We computed the idFV715 model sensitivity of acetate production according to,

$$V^R{}_A = \frac{A_{ij} - \hat{A}_j}{\hat{A}_j} \cdot 100 \quad (2)$$

where $\hat{A}_j$ are the acetate concentrations given by the model in the base case at time j, and A_{ij} are the acetate concentrations given by the model at time j in the ith disturbed case. The latter included variations in initial concentrations of sugar and nitrogen, and fermentation temperature. The following table summarizes the respective sensitivities.

Table 2: idFV715 model sensitivities of acetate concentrations to variations in initial concentrations of sugar and nitrogen, and fermentation temperature. Final fermentation points were used to this analyses.

			Relative acetate synthesis in model [%]
Sugar (g/L)	198	-1%	-32.9
	202	+1%	5.5
Nitrogen (mg/L)	297	-1%	-41.1
	303	+1%	-9.9
Temperature (°C)	27.7	-1%	20.2
	28.3	+1%	-2.3

The table shows that small variations in sugar, nitrogen and temperature have a strong impact in acetate simulations.
