# Supplementary material for: Expanding a dynamic flux balance model of yeast fermentation to genome-scale
Source: BMC Syst Biol. 2011 May 19;5:75. doi: 10.1186/1752-0509-5-75 (PMC3118138; doi:10.1186/1752-0509-5-75)
Supplement: Additional file 2 — Kinetic expression used in the idFV715 model. This file includes kinetic expression used in the idFV715 model. Sugar, nitrogen and maintenance expressions are detailed. [file 1752-0509-5-75-S2.PDF]

Additional File 2

1. Sugar uptake

$$v'_{S,i} = \frac{v_{\max,i} \cdot S}{\left(1+\frac{E}{K_E}\right)\cdot\left(K_{S,i}\cdot\left(1+\frac{S}{K_{S,i}}\right)+S\right)} \tag{1}$$

where $v'_{S,i}$ are either glucose or fructose partial uptake rates for each of the 5 main hexose transporters i, expressed under anaerobic conditions [1]; $v_{\max,i}$ and $K_{S,i}$ are the maximum uptake rate and sugar saturation constant for the ith transporter; and K_E is the ethanol inhibition constant (Table S1). Hexose transporters are active or inactive depending on the growth phase and glucose concentration in the medium. The uptake rates for glucose or fructose, $v_{S,i}$, are given by the weighted average of the uptake rates of the active transporters (Eq. 2).

$$v_{S,i} = f \cdot \sum_i \alpha_i v'_{S,i} \tag{2}$$

where f is an empirical temperature-dependent efficiency factor for sugar transport that ranges from 0 to 1 [2].

Table S1: Kinetic parameters used for modeling sugar uptake rates [2]. The expression of hexose transporters during fermentation was set using weights, α, corresponding to the relative abundance of each transporter in the given growth phase: $\alpha = 0.5$ for HXT1 and HXT3 in the exponential phase; $\alpha = 1$ for HXT3 from onset stationary phase until [glucose] < 20g/L; $\alpha = 0.5$ for HXT3 and $\alpha = 0.25$ for HXT6 and HXT7 during the stationary phase.

Transporter	V_{max} [ggDCW^{-1}h^{1}]	K_g [gL^{1}]	K_f [gL^{1}]
HXT1	7.45	18	54
HXT3	2.7	10.8	22.3
HXT6	1.31	0.18	0.47
HXT7	1.31	0.36	0.83

2. Nitrogen uptake

$$v_{N,i} = \left(a\tan\left(\frac{n-0.2}{7.25e^{-3}} \right) \cdot \Pi + \frac{1}{2} \right) \cdot \frac{3e^{-4}}{N_i \cdot C_i} \tag{3}$$

Here, n represents total nitrogen content in the medium, N_i is the number of nitrogen atoms in the ith nitrogen-containing substrate, and C_i is the nitrogen-containing substrate ith with a concentration above 0.1mg/L in the extracellular medium.

3. Maintenance

$$v_{mATP} = \left[\left(0.17 \cdot N_{in} + 0.09 \right) + \left(\frac{0.26}{e^{\left(\frac{31.17-E}{7.3} \right)}} \right) \right] \cdot v_{S,i} \tag{4}$$

Here, N_{in} represents the initial nitrogen concentration (i.e. summed across all N-compounds); $v_{S,i}$ is the sugar uptake rate and E is the ethanol concentration in g/L.

4. Carbohydrates accumulation

$$v_{CARB,i} = e^{-\left(4.5+18\cdot S^3+1.47\cdot e^S\right)} \tag{5}$$

This function defines an UB in the LP. S represents the remaining sugar in the medium.

5. Biomass equation

			Previous model Nitrogen limited	iFF708 Carbon limited	iIN800 Carbon limited	iIN800 nitrogen limited	IFV715 Carbon limited	IFV715 nitrogen limited
Amino acids			0.018287					
	L-Alanine	ALA		0.4590	0.3570	0.2520	0.3570	0.2520
	L-Arginine	ARG		0.1610	0.1360	0.0980	0.1360	0.0980
	L-Asparagine	ASN		0.1020	0.1720	0.1530	0.1720	0.1530
	L-Aspartate	ASP		0.2980	0.1720	0.1530	0.1720	0.1530
	L-Cysteine	CYS		0.0070	0.0430	0.0440	0.0430	0.0440
	L-Glutamate	GLU		0.3020	0.2680	0.2310	0.2680	0.2310
	L-Glutamine	GLN		0.1050	0.2680	0.2310	0.2680	0.2310
	Glycine	GLY		0.2900	0.3250	0.2780	0.3250	0.2780
	L-Histidine	HIS		0.0660	0.0750	0.0710	0.0750	0.0710
	L-Isoleucine	ILE		0.1930	0.1720	0.1420	0.1720	0.1420
	L-Leucine	LEU		0.2960	0.2500	0.2070	0.2500	0.2070
	L-Lysine	LYS		0.2860	0.2390	0.2040	0.2390	0.2040
	L-Methionine	MET		0.0510	0.0500	0.0440	0.0500	0.0440
	L-Phenylalanine	PHE		0.1340	0.1140	0.0920	0.1140	0.0920
	L-Proline	PRO		0.1650	0.1290	0.1180	0.1290	0.1180
	L-Serine	SER		0.1850	0.2540	0.2250	0.2540	0.2250
	L-Threonine	THR		0.1910	0.1970	0.1600	0.1970	0.1600
	L-Tryptophan	TRP		0.0280	0.0270	0.0280	0.0270	0.0280
	L-Tyrosine	TYR		0.1020	0.0960	0.0680	0.0960	0.0680
	Valine	VAL		0.2650	0.0000	0.0000	0.0000	0.0000
Carbohydrates			0.0154					
	Glycogen	GLYCOGEN		0.5190	0.5190	0.6670	0.5190	0.6670
	alpha,alpha-Trehalose	TRE		0.0234	0.0230	0.0850	0.0230	0.0850
	Mannan	MANNAN		0.8080	0.8210	0.9940	0.8210	0.9940
	1,3-beta-D-Glucan	13GLUCAN		1.1348	1.1360	0.9630	1.1360	0.9630
RNA			0.001651					
	AMP	AMP		0.0460	0.0510	0.0400	0.0510	0.0400
	GMP	GMP		0.0460	0.0510	0.0400	0.0510	0.0400
	CMP	CMP		0.0450	0.0500	0.0390	0.0500	0.0390
	UMP	UMP		0.0600	0.0670	0.0520	0.0670	0.0520
DNA			0.000161					
	dAMP	DAMP		0.0040	0.0040	0.0040	0.0040	0.0040
	dCMP	DCMP		0.0020	0.0020	0.0030	0.0020	0.0030
	dTMP	DTMP		0.0040	0.0040	0.0040	0.0040	0.0040
	dGMP	DGMP		0.0020	0.0020	0.0030	0.0020	0.0030
Lipids			0.001456					
	Phosphatidylcholine	PC		0.00600	0.002884	0.001660	0.00309	0.00238
	1-Phosphatidyl-D-myo-inositol	PINS		0.00500	0.001531	0.001656	0.00153	0.00166
	Phosphatidylserine	PS		0.00200	0.000373	0.000302	0.00037	0.00030
	Phosphatidylethanolamine	PE		0.00400	0.000697	0.000083	0.00070	0.00008
	Acyl_acids	ACYL			0.000206	0.000723		
	Triacylglycerol	TAGLY		0.00700	0.000781	0.003618	0.00078	0.00362
	Ergosterol-ester	ERGOSTE			0.000812	0.004632		
	Ergosta-5,7,22,24(28)-tetraenol	ERTEOL			0.000125	0.000167	0.00013	0.00017
	Ergosterol	ERGOST		0.00100	0.005603	0.005155	0.00642	0.00532
	Zymosterol	ZYMST		0.00200	0.000015	0.000051	0.00002	0.00005
	Episterol	EPST			0.000096	0.000062	0.00010	0.00006
	Fecosterol	FEST			0.000114	0.000068	0.00011	0.00007
	Lanosterol	LNST			0.000032	0.000074	0.00003	0.00007
	4,4-Dimethylzymosterol	DMZYMST			0.000056	0.000046	0.00006	0.00005
	Ceramide-I	CER1			0.000351	0.000075		
	Ceramide-II	CER2			0.000066	0.000009	0.00042	0.00008
	Phosphatidate	PA		0.00100	0.000000	0.000000	0.00100	0.00000
	Sulfate	SLF		0.02000			0.02000	0.02000
	ATP	ATP		59.27600			59.276001	59.276001

Due to the unavailability of some lipid intermediates, some compounds were condensed into only one; Phosphatidylcholine and Acyl acids, ergosterol and ergosterol-ester and ceramide I and ceramide II were grouped into three compounds.

[1] Perez M, Luyten K, Michel R, Riou C, Blondin B: **Analysis of Saccharomyces cerevisiae hexose carrier expression during wine fermentation: both low- and high-affinity Hxt transporters are expressed.** *FEMS Yeast Res* 2005, **5:**351-361.
[2] Pizarro F, Varela C, Martabit C, Bruno C, Perez-Correa JR, Agosin E: **Coupling kinetic expressions and metabolic networks for predicting wine fermentations.** *Biotechnol Bioeng* 2007, **98:**986-998.
