# Supplementary material for: Expanding a dynamic flux balance model of yeast fermentation to genome-scale
Source: BMC Syst Biol. 2011 May 19;5:75. doi: 10.1186/1752-0509-5-75 (PMC3118138; doi:10.1186/1752-0509-5-75)
Supplement: Additional file 3 — Model validation. This file includes tables where results using previous DFBA model can be directly compared to current model results of Table 4. Also, a description of metabolic engineering conditions was included. [file 1752-0509-5-75-S3.PDF]

Additional File 3

1. Fermentation performances of our previous published DFBA model.

Fermentation profiles prediction of our previous DFBA model under different medium conditions. Twenty laboratory and ten industrial fermentations, with approx. 25 samples per fermentation, were analyzed. Performance is expressed as percentage of correlation between experimental and model data. Both, initial conditions and data sets to compare were equal to which were used to elaborate table 4 in the main document. Therefore numbers in both tables are comparables.

Initial Conditions		Lab fermentations					Industrial fermentations
Nitrogen	Sugar	Sugar uptake	Nitrogen uptake	Ethanol	Glycerol	Biomass	Sugar uptake
Low 50-200 mg/L	Low 100-200 g/L	99.8	97.0	99.8	99.5		
	High 201-350 g/L	99.8		99.5	99.5	93.0	
High 201-540 mg/L	Low 100-200 g/L	97.3	97.1	97.0	95.0		97.9
	High 201-350 g/L	99.5	98.2	98.0	98.8	95.5	98.7

To simplify the comparison of model idFV715 with our previous DFBA model, data contained in Table 4 and the table above was summarized in the following table.

		idFV715	Reduced DFBA model
Sugar uptake	95 % error interval [%]	1.57	1.51
	R2	98.62	98.67
Nitrogen uptake	95 % error interval [%]	0.56	0.54
	R2	98.30	97.43
Metabolite synthesis	95 % error interval [%]	3.86	3.43
	R2	96.09	97.56

Here we see that the 95% average confidence intervals and average correlation factors were quite similar, indicating that both models have basically the same predictive capability.

2. Product prediction under genetic and environmental disturbances.

This section provides additional information regarding Figure 3. 35 environmental/genetic disturbances were reproduced by idFV715 model. The strategies were extracted from 9 different papers representing deletions, overexpressions and insertions in single, double and up to triple strategies at the same time.

Simulations were carried out trying to be as close as possible to literature environmental/genetic conditions. It was simulated MS, MS300, MM and YPD media. All cases were anaerobic conditions. Initial content of N-compounds were added individually. Temperatures were in the range 26-30 degrees Celsius and a variety of strains were used. Currently idFV715 does not offer a factor correction for different strains but future model modifications could include the use of different nitrogen and glucose uptake factors that reflect strain differences.

Numbers in the results section of the table below show the relative changes between model performance and experimental results for all 35 experiments. For example simulation #1 showed 2%, -22% and -15% of error in the prediction of ethanol, glycerol and acetate, respectively, for an ald6 mutation in that specific environmental condition.

#	Parameters					Genes modified (deleted, overexpressed)	Reference	Percent Difference between idFV715 Model and Experimental Results			
	Glucose	Amino acid (mg/L)	Amonnia (mg/L)	Temp Celsius	Strain			Biomass	Ethanol	Glycerol	Acetate
1	200	340	120	26	VL1	ald6	Cambon 2006	-	2.19	-22.40	-15.47
2	200	340	120	26	VL1	GPD1	Cambon 2006	-	8.84	-46.75	-268.09
3	200	340	120	26	VL1	GPD1 / ald6	Cambon 2006	-	15.99	-164.76	696.99
4	200	340	120	26	K1M	ald6	Cambon 2006	-	1.25	-25.79	-8.45
5	200	340	120	26	K1M	GPD1	Cambon 2006	-	3.38	8.49	-13.64
6	200	340	120	26	K1M	GPD1 / ald6	Cambon 2006	-	8.76	-135.01	597.47
7	200	340	120	26	BC	ald6	Cambon 2006	-	3.73	-7.16	4.70
8	200	340	120	26	BC	GPD1	Cambon 2006	-	3.09	74.40	-162.98
9	200	340	120	26	BC	GPD1 / ald6	Cambon 2006	-	13.67	-157.84	626.48
10	20	-	5000	30	ANGA	gpd1	Peng Guo 2009	5.00	-14.00	-4.45	-
11	20	-	5000	30	ANGA	gpd2	Peng Guo 2009	7.20	-19.33	0.10	-
12	80	340	120	28	s288c	ald6	Eglington 2002	-	9.63	-22.53	-29.47
13	80	340	120	28	s288c	GPD1	Eglington 2002	-	18.41	57.58	424.02
14	200	180	120	26	R	GPD1	Remize 1999	14.05	3.80	12.00	-70.44
15	200	180	120	28	V5	ald6	Remize 2000	-	-	-26.62	-23.52
16	200	180	120	28	V5	ald6/ald4	Remize 2000	-	-	-113.68	-26.83
17	240	340	120	28	V5	ald6	Dequin 2009	-10.26	9.20	-77.48	-7.54
18	240	340	120	28	V5	GPD1 / ald6	Dequin 2009	27.42	-10.05	-5.06	-22.04
19	240	340	120	28	V5	GPD1 / BDH1 / ald6	Dequin 2009	36.10	-15.66	94.44	527.86
20	240	340	120	28	V5	GPD1 / BDH1 site directed/ ald6	Dequin 2009	27.76	-8.43	-8.02	103.53
21	200	340	120	28	V5	ald6	Dequin 2009	-	1.57	-34.44	-
22	200	340	120	28	V5	GPD1 / ald6	Dequin 2009	-	-5.24	6.20	-
23	200	340	120	28	V5	GPD1 / BDH1 / ald6	Dequin 2009	-	-17.02	102.16	-
24	200	340	120	28	V5	GPD1 / BDH1 site directed/ ald6	Dequin 2009	-	-8.98	-25.12	-
25	200	180	120	28	V5	ald4	Saint Prix 2004	-	-	-	523.16
26	200	180	120	28	V5	ald5	Saint Prix 2004	-	-	-	266.51
27	200	180	120	28	V5	ald6	Saint Prix 2004	-	-	-	-626.48
28	200	180	120	28	V5	ald2/3	Saint Prix 2004	-	-	-	0.00
29	50	180	120	28	CEN.PK	ald4	Saint Prix 2004	-	-	-	90.67
30	50	180	120	28	CEN.PK	ald5	Saint Prix 2004	-	-	-	21.43
31	50	180	120	28	CEN.PK	ald6	Saint Prix 2004	-	-	-	-23.80
32	50	180	120	28	CEN.PK	ald2/3	Saint Prix 2004	-	-	-	126.56
33	200	180	120	28	CEN.PK	ald5	Saint Prix 2004	-	-	-	21.43
34	20	-	23000	30	DC124	fps1	Zhang 2006	-	-0.17	0.00	-
35	20	-	5000	30	CEN.PK	gpd1/gpd2/MHPF	Guadalupe 2010	-	7.45	0.00	74.45
